# Association of Preterm Birth With Attention-Deficit/Hyperactivity Disorder–Like and Wider-Ranging Neurophysiological Impairments of Attention and Inhibition

**DOI:** 10.1016/j.jaac.2016.10.006

**Published:** 2017-01

**Authors:** Anna-Sophie Rommel, Sarah-Naomi James, Gráinne McLoughlin, Daniel Brandeis, Tobias Banaschewski, Philip Asherson, Jonna Kuntsi

**Affiliations:** a Medical Research Council Social, Genetic and Developmental Psychiatry Centre, the Institute of Psychiatry, Psychology and Neuroscience, King’s College London, London, UK; b Central Institute of Mental Health, Medical Faculty Mannheim/Heidelberg University, Mannheim, Germany; c Psychiatric Hospital, University of Zurich, Zurich, Switzerland; the Center for Integrative Human Physiology, University of Zurich, and the Neuroscience Center Zurich, University of Zurich

**Keywords:** ADHD, preterm birth, EEG, event-related potential, neurocognitive impairment

## Abstract

**Objective:**

Preterm birth has been associated with an increased risk of attention-deficit/hyperactivity disorder (ADHD)–like symptoms and cognitive impairments similar to those seen in ADHD, including attention and inhibitory control difficulties. Yet data on direct comparisons across ADHD and preterm birth on cognitive-neurophysiological measures are limited.

**Method:**

We directly compared 186 preterm-born adolescents to 69 term-born adolescents with ADHD and 135 term-born controls on cognitive-performance and event-related potential measures associated with attentional and inhibitory processing from a cued continuous performance test (CPT-OX), which we have previously shown to discriminate between the adolescents with ADHD and controls. We aimed to elucidate whether the ADHD-like symptoms and cognitive impairments in preterm-born individuals reflect identical cognitive-neurophysiological impairments in term-born individuals with ADHD.

**Results:**

Go-P3 amplitude was reduced, reflecting impaired executive response control, in preterm-born adolescents compared to both controls and adolescents with ADHD. Moreover, in preterm-born adolescents, as in term-born adolescents with ADHD, contingent negative variation amplitude was attenuated, reflecting impairments in response preparation compared to controls. Although the ADHD group showed significantly increased NoGo-P3 amplitude at FCz compared to preterm group, at Cz preterm-born adolescents demonstrated significantly decreased NoGo-P3 amplitude compared to the control group, similar to term-born adolescents with ADHD.

**Conclusion:**

These findings indicate impairments in response preparation, executive response control, and response inhibition in preterm-born adolescents. Although the response preparation and response inhibition impairments found in preterm-born adolescents overlap with those found in term-born adolescents with ADHD, the preterm group also shows unique impairments, suggesting more wide-ranging impairments in the preterm group compared to the ADHD group.

In the developed world, 8.6% of individuals are born preterm (i.e., before 37 completed weeks of gestation).^1^ Although survival rates of preterm-born individuals have increased greatly,^2^, ^3^ preterm birth is associated with the risk of adverse long-term outcomes.^4^, ^5^ A meta-analysis demonstrated that preterm-born children (n = 1,556) were at heightened risk (relative risk [RR] = 2.64) for developing attention-deficit/hyperactivity disorder (ADHD) relative to controls (n = 1,720).^4^ A population-based study of Norwegian adults further reported a 1.3- and 5-fold increased risk for ADHD in adults born preterm (<37 weeks) and extremely preterm (<28 weeks), respectively.^6^

ADHD is characterized by developmentally inappropriate and impairing levels of hyperactivity, impulsivity, and/or inattention.^7^ A meta-analysis in children with ADHD showed impairments in sustained attention (indexed by increased numbers of omitted responses or omission errors), impairments in response inhibition (indexed by increased numbers of error responses or commission errors), and intraindividual fluctuations in reaction times (reaction time variability [RTV]) compared to controls.^8^ In adults with ADHD, similar impairments have been reported.^9^, ^10^, ^11^, ^12^ A cognitive profile that resembles that of individuals with ADHD, including impairments in attention and inhibitory control, is also frequently associated with preterm birth.^13^ A meta-analysis of nine studies found that teacher- and parent-rated attention problems in very preterm children (<33 weeks’ gestation) were 0.43 to 0.59 standard deviations higher than for controls, respectively.^13^ Furthermore, impairments in response inhibition have been found in children^14^ and young adults^15^ born preterm.

Although research has provided strong support for the link between preterm birth and ADHD, little is known about the underlying risk pathways. Cognitive performance data alone provide only indirect insight into covert processing as various covert mechanisms may result in indistinguishable overt performance on cognitive tasks. The study of event-related potentials (ERPs), which are electrical potentials generated by the brain in response to internal or external events such as stimuli and responses,^16^ allows direct investigation of covert brain processes with millisecond temporal resolution.^17^, ^18^ ERP measures permit a sensitive comparison of the cognitive-neurophysiological profiles associated with preterm birth and ADHD, enabling us to investigate whether the symptoms and impairments seen in individuals born preterm are identical to those associated with ADHD or whether they are part of more wide-ranging impairments. Consequently, neurophysiological assessments have the potential to further elucidate the risk pathways underlying preterm birth and ADHD.

Although countless ERP studies have been conducted in preterm-born infants in neonatal intensive care units^19^, ^20^ and in the postnatal period,^21^, ^22^, ^23^ less ERP research has been carried out in children, adolescents, or adults born preterm. The ERP studies that have been conducted in preterm-born children have focused mainly on auditory ERP components.^24^, ^25^, ^26^ Auditory ERP components are neurophysiological correlates of cortical sound processing and sound discrimination: they are generated involuntarily by the brain during basic auditory encoding (indexed by the ERP component P1) or as a response to a perceived change in continuously repeated sounds (indexed by the ERP components N2, mismatch negativity [MMN] and P3a).^16^ Responses to novel sounds (as indexed by N2, MMN, and P3a amplitudes) are hypothesized to reflect an individual’s capacity to allocate attention.^16^, ^25^ Auditory ERPs are therefore useful to study attention in participants who cannot easily respond behaviorally, such as young infants and children. In auditory ERP studies, increased N2 amplitudes, interpreted as reflecting impaired attention orienting, have been found in children born very preterm (<32 weeks).^24^, ^26^ Preterm-born children have also demonstrated abnormalities in other early sensory and attentional ERP components (MMN, P1 and P3a).^24^, ^25^, ^26^ Despite this initial evidence for impairments in ERP measures of attentional processing in preterm-born individuals, the research overall is limited, and no studies of preterm birth to date have investigated ERP components that sensitively capture the attentional and inhibitory impairments seen in individuals with ADHD.

One of the most common cognitive tasks used to study attentional processing and response inhibition in individuals with ADHD is the cued continuous performance test (CPT-OX). The CPT-OX requires participants to detect target stimuli among a sequence of distractor stimuli. Omission errors (OE; the lack of a response to a target) are assumed to represent impairments in sustained attention, whereas commission errors (CE; responses to distractor stimuli) are an index of response inhibition. ERPs associated with the CPT are the Go-P3, which is an electrical potential generated by the brain in response to the target stimulus, the NoGo-P3, which is an electrical potential generated by the brain in response to the distractor stimulus and reflects response inhibition, as well as the Cue-P3 and contingent negative variation (CNV), which occur in response to the cue stimulus and are thought to reflect attentional orienting to a cue and motor response preparation respectively. CPT performance is typically impaired in individuals with ADHD, who usually demonstrate increased reaction time variability, a greater number of OEs, and a greater number of CEs.^8^ In addition, ERP studies using the CPT-OX have found that individuals with ADHD also show impaired response inhibition, response preparation, and attentional orienting as indexed by reduced NoGo-P3, contingent negative variation (CNV), and Cue-P3 amplitudes.^27^, ^28^, ^29^ Yet, not all studies have reported case−control differences in Cue-P3 amplitude.^30^, ^31^ Although some studies report attenuated Go-P3 amplitude in individuals with ADHD,^32^, ^33^, ^34^ indexing impaired executive response control, others show no case-control differences.^27^, ^28^, ^35^ Finally, case-control differences in N2 amplitude in response to distractor stimuli (NoGo-N2) are typically not found in the CPT-OX.^28^, ^31^, ^36^

Direct comparisons on ERP measures between preterm-born individuals and term-born individuals with ADHD are scarce. One ERP study investigated attentional processing in very-low-birthweight children born preterm (<1,501 g and <34 weeks) with and without ADHD, as well as in term-born controls and term-born individuals with ADHD.^37^ Term- and preterm-born children with ADHD, who showed increased mean reaction time (MRT), RTV, and a greater number of commission and omission errors on a visual oddball paradigm, also demonstrated an increased NoGo-N2 amplitude compared to term-born controls and preterm-born participants without ADHD. However, the sample size was small (n = 41 across four groups). No study to date has compared ERP components associated with attentional and inhibitory processing in both ADHD and preterm birth using a detailed measure of attention and inhibitory control such as the CPT-OX.

We previously reported findings on ADHD case−control differences on cognitive and neurophysiological markers of ADHD. With the use of the CPT-OX, we demonstrated that sustained attention (indexed by omission errors), response inhibition (indexed by commission errors and NoGo-P3 amplitude), intraindividual fluctuations in reaction times (RTV), response preparation (indexed by CNV amplitude), and attentional orienting (indexed by Cue-P3 amplitude) successfully discriminated between adolescents with ADHD and controls.^38^ Conflict monitoring as indexed by NoGo-N2 amplitude was not previously investigated in this sample of ADHD and control adolescents.^38^ Having established the cognitive-performance and ERP measures that sensitively capture the attentional and inhibitory impairments in adolescents with ADHD, we now compare new data obtained from adolescents born preterm on identical measures to the data previously obtained from the ADHD and control participants.

In the current study, we therefore aim to establish whether the cognitive impairments associated with preterm birth, including attention and inhibitory control difficulties, reflect neurophysiological impairments identical to those observed in term-born individuals with ADHD.

## Method

### Sample

The sample consisted of 186 preterm-born participants (41 sibling pairs, 104 singletons), 69 participants with ADHD (4 sibling pairs, 61 singletons) and 135 controls (61 sibling pairs, 13 singletons). The groups differed significantly in terms of age, IQ, sex distribution, gestational age (GA), and ADHD symptom scores (Table 1). The ADHD group showed significantly higher ADHD symptoms and functional impairment than both the preterm group (*t* = −16.55, df = 178, *p* < .001, *d* = 2.53; *t* = −17.23, df = 178, *p* < .001, *d* = 2.94 respectively) and control group (*t* = 20.06, df = 134, *p* < .001, *d* = 3.74; *t* = 19.70, df = 134, *p* < .001, *d* = 3.72 respectively). The preterm group further demonstrated significantly higher ADHD symptoms and functional impairment than the control group (*t* = 4.71, df = 213, *p* < .001, *d* = 0.53; *t* = 3.83, df = 213, *p* < .001, *d* = 0.45 respectively). Although only 4% of the preterm participants were treated with stimulant medication, 47% of the participants with ADHD were treated with stimulant medication at the time of the assessment. A 48-hour ADHD medication-free period was required before assessments. Written informed consent was obtained following procedures approved by the London-Surrey Borders Research Ethics Committee (09/H0806/58) and the National Research Ethics Service Committee London—Bromley (13/LO/0068).

**Table 1:** Characteristics of Study Participants

	ADHD n = 69	Preterm n = 186	Control n = 135	Statistic	*df*	*p* Value
				—	—	—
GA, wk (SD)	39.9 (1.4)	33.0 (3.0)	39.9 (1.3)	*t* = –23.0	253	<.001
GA range, wk	3,742	24–36	37–43	—	—	—
IQ (SD)	97.7 (13.8)	104.7 (12.3)	110.4 (12.2)	*t* = –3.2	253	.002
Age (SD), y	18.5 (3.0)	14.9 (1.9)	17.8 (2.1)	*t* = –12.0	253	<.001
Age range, y	12.7–25.9	11.0–20.0	11.9–21.6	—	—	—
Males, %	88.4	54.3	75.6	*t* = 4.6	253	<.001
Conners parent-rated ADHD symptom score (SD)	35.8 (10.6)	11.2 (9.4)	7.0 (5.6)	*t* = 1.97	253	.050
BFIS score (SD)	16.4 (5.4)	3.7 (4.1)	2.1 (2.5)	*t* = –2.23	253	.027

Note: ADHD = attention-deficit/hyperactivity disorder; BFIS = Barkley Functional Impairment Scale; GA = gestational age.

The preterm group was recruited from secondary schools in Southeast England. All preterm participants had one full sibling available for ascertainment and were born before 37 weeks’ gestation. Siblings of preterm-born individuals were included in the preterm group if they were also born preterm (before 37 weeks’ gestation), to maximize the number of participants in the preterm group. Term-born siblings of preterm-born individuals were not included in this analysis. Most preterm-born participants were of European white descent (84.6%). The other ethnicities represented were British Asian (3.7%), mixed white and black Caribbean (2.1%), mixed white and British Asian (1.6%), Indian (1.1%), mixed white and Indian (1.1%), black Caribbean (0.5%), mixed black and British Asian (0.5%), and other (2.7%). Seven individuals from the preterm sample were excluded because medical birth records could not corroborate preterm status (GA ≥37 weeks). One individual was excluded because of an IQ of <70. Eight preterm-born individuals met diagnostic criteria for a research diagnosis of ADHD. Since here preterm birth is investigated as a potential risk factor for ADHD, preterm-born individuals who demonstrated high levels of ADHD symptoms were not excluded from the analysis (for the descriptive statistics and the analysis of the sample without preterm-born individuals who met a research diagnosis for ADHD, see Supplement I and Table S1, available online).

ADHD and control sibling pairs who had taken part in our previous research^39^, ^40^ were invited to take part in a follow-up study.^38^ All participants were of European white descent and had one full sibling available for ascertainment. Participants with ADHD and their siblings were included in the ADHD group if they had a clinical diagnosis of *DSM-IV* combined-type ADHD during childhood and met *DSM-IV* criteria for any ADHD subtype at follow-up. Siblings of individuals with ADHD who did not meet *DSM-IV* criteria for any ADHD subtype at follow-up were not included in this analysis. The control group was initially recruited from primary (aged 6–11 years) and secondary (aged 12–18 years) schools in the United Kingdom, aiming for an age and sex match with the ADHD sample. Control individuals and their siblings were included in the control group if they did not meet *DSM-IV* criteria for any ADHD subtype either in childhood or at follow-up.

Exclusion criteria for all groups were IQ of <70, general learning difficulties, cerebral palsy or any other medical condition that affects motor coordination including epilepsy, as well as brain disorders and any genetic or medical disorder that might mimic ADHD. In addition, preterm birth was an exclusion criterion in the ADHD and control groups, because this study aimed to establish whether the cognitive impairments associated with preterm birth reflect identical neurophysiological impairments in term-born individuals with ADHD.

We followed up the sample on average 5.8 years (SD = 1.1) after initial assessments. The ADHD and control groups were previously included in a study investigating ADHD case−control differences on cognitive and neurophysiological markers of ADHD persistence and remission.^38^ Although ADHD−control differences for this sample have been reported previously, here the ADHD and control groups are compared to a group of preterm-born adolescents.

At follow-up, six participants from the ADHD−sibling pair sample were excluded from the group analyses because of missing parent ratings of clinical impairment. Therefore, their current ADHD status could not be determined. Two additional participants from the ADHD−sibling pair sample were excluded because of drowsiness during the cognitive task. Two participants with childhood ADHD, who did not meet ADHD symptom criteria but did meet clinical levels of impairment at follow-up, were excluded to minimize heterogeneity in the ADHD sample. In addition to these exclusions, which are identical to those in our previous analysis,^38^ we also excluded six participants from the ADHD−sibling pair sample who were born preterm, as well as 12 individuals from the ADHD−sibling pair sample who provided no information on GA.

Six control participants were removed from the analyses for meeting *DSM-IV* ADHD criteria based on the parent-rated Barkley Informant Rating Scale.^41^ In addition to these exclusions, which are identical to those in our previous analysis,^38^ we also excluded 37 participants from the control−sibling pair sample because no GA information was available.

### Measures

#### Diagnostic Interview for ADHD in Adults

The Diagnostic Interview for ADHD in Adults (DIVA)^42^ is a semi-structured interview designed to evaluate the *DSM-IV* criteria for both adult and childhood ADHD symptoms and impairment. It consists of 18 items used to define the *DSM-IV* symptom criteria for ADHD. Each item is scored affirmatively if the behavioral symptom was present often within the past 6 months.

#### Conners’ Parent Rating Scale

Inattentive and hyperactive-impulsive symptoms were measured using the Long Version of Conners’ Parent Rating Scale.^43^ Summing the scores on the nine-item hyperactive-impulsive and nine-item inattentive *DSM-IV* symptoms subscales forms a total *DSM-IV* ADHD symptoms subscale.

#### Barkley Functional Impairment Scale

The Barkley Functional Impairment Scale (BFIS)^41^ is a 10-item scale used to assess the levels of functional impairments commonly associated with ADHD symptoms in five areas of everyday life: family/relationship; work/education; social interaction; leisure activities; and management of daily responsibilities. Each item ranged from 0 (never or rarely) to 3 (very often).

In the preterm and ADHD groups, ADHD was assessed using parental ADHD symptom ratings on the DIVA and the BFIS for all participants, for consistency. If participants were usually on stimulant medication, parents were instructed to consider their children’s ADHD symptoms off medication. A research diagnosis of ADHD was made if participants scored six or more on either the inattention or hyperactivity-impulsivity subscales of the DIVA and if they received two or more positive scores on two or more areas of impairment on the BFIS. In the control group, ADHD was assessed using parental ADHD symptom ratings on the BFIS for all participants, for consistency. Control participants were excluded from the analysis if they received two or more positive scores on two or more areas of impairment on the BFIS.

#### IQ

The vocabulary and block design subtests of the Wechsler Abbreviated Scale of Intelligence− Fourth Edition (WASI-IV)^44^ were administered to all participants to derive estimates of IQ.

#### Cued Continuous Performance Test

The CPT-OX is a cued Go/NoGo task that probes attention, preparation, and response inhibition. The task consisted of 400 black letter arrays made up of a center letter and incompatible flankers on each side to increase difficulty. The presented arrays included the cue letter O, the target letter X, as well as the distractors H, B, C, D, E, F, G, J, and L. Cue and target letters (O and X respectively) were flanked by incompatible letters (XOX and OXO respectively). Participants were instructed to ignore the flanking letters and respond as quickly as possible to cue-target sequences (O-X). A total of 80 cues (XOX) were followed by the target (OXO) in 40 trials (Go condition), and by neutral distractors in the remainder of trials (NoGo condition). In 40 trials, the target letter X was not preceded by a cue O and had to be ignored. Letters were presented every 1.65 seconds for 150 milliseconds in a pseudo-randomized order. Ten practice trials preceded the main task and were repeated, if required, to ensure participant comprehension. Participants were instructed to respond only to Cue-Go sequences by pressing a button as quickly as possible with the index finger of their preferred hand. Participants were further asked to withhold the response in the presence of a NoGo stimulus, in the presence of a Go stimulus not preceded by a cue, or in the presence of any other irrelevant letters. Task duration was 11 minutes.

Cognitive-performance measures obtained from the CPT-OX include MRT (mean latency of response after target onset in milliseconds), RTV (standard deviation of target reaction time), and number of errors. MRT and RTV were obtained from correct Go trials. Errors included total omission errors (OE; nonresponses to Go trials) and total commission errors (CE; responses to Cue, NoGo, or distractor stimuli).

### Procedure

Participants attended a single 4.5-hour research session, which included an EEG assessment, an IQ test, and clinical interviews. As part of the EEG assessment, participants completed a CPT with flankers (CPT-OX).^45^ The task was preceded by two 3-minute resting-state recordings and was the first of three cognitive EEG tasks to be conducted during the testing session.

### Electrophysiological Recording and Analysis

The EEG was recorded from a 62-channel direct-current coupled (DCC) recording system (extended 10–20 montage), using a 500-Hz sampling-rate, impedances less than 10 kΩ, and FCz as the recording reference. The electro-oculograms were recorded from electrodes above and below the left eye and at the outer canthi. The EEG data were analyzed using Brain Vision Analyzer 2.0 (Brain Products, Germany). Raw EEG recordings were down-sampled to 256 Hz, re-referenced to the average of all electrodes, and digitally filtered using Butterworth band-pass filters (0.130Hz, 24 dB/oct). All trials were also visually inspected for electrical artifacts or obvious movement, and sections of data containing artifacts were removed manually. Ocular artefacts, corresponding to blink-related, vertical and horizontal eye movements, were identified using the infomax Independent Component Analysis (ICA) algorithm,^46^ which allows for removal of the components associated with ocular artifacts by back-projection of all but those components. Sections of data with remaining artifacts exceeding ±100 μV in any channel or with a voltage step greater than 50 μV were automatically rejected.

For the CPT, stimulus-locked epochs (stimulus window from −200 to 1,650 ms) were averaged based on three different response conditions: Cue, Go, and NoGo. Averages were calculated for trials with correct responses (Go) or correctly rejected trials (NoGo and Cue), which included at least 20 artifact-free segments (for the number of artifact-free segments per trial, see Table 2). Based on previous research,^27^, ^28^, ^47^ ERP measures were identified within selected electrodes and latency windows for which effects were expected to be largest. These measures were then confirmed separately for the three groups using topographic maps.

**Table 2:** Artifact-Free Segments per Trial in the Cued Continuous Performance Test

Trial	ADHD Mean (SD)	Preterm Mean (SD)	Control Mean (SD)
Cue	65.2 (6.6)	63.5 (7.0)	68.1 (6.0)
Go	34.3 (4.5)	33.9 (4.2)	36.4 (3.3)
NoGo	29.1 (3.4)	28.6 (3.1)	30.7 (3.1)

Note: Means and standard deviations (SD) for the attention-deficit/hyperactivity disorder (ADHD), preterm, and control group are shown.

In Cue trials, the P3 was measured at Pz between 300 and 650 milliseconds, and the CNV was measured at Cz and CPz between 1,300 and 1,650 milliseconds. In Go trials, the P3 was measured at CPz and Pz between 250 and 500 milliseconds. No clear N2 was observed in Go trials, consistent with other studies using tasks with low conflict-monitoring demands^31^, ^48^ and was therefore not included in the analysis. In NoGo trials, the P3 was measured at FCz and Cz between 250 and 550 milliseconds and the N2 was measured at Fz between 175 and 325 milliseconds.

Whereas ERP components with baseline correction are thought to represent the absolute change in neural activity elicited by the stimulus, ERP components without baseline correction are thought to reflect the absolute state of neural activity measured at a given time.^49^ Here, results are presented and interpreted without baseline correction, as most previous ERP analyses on CPT-OX did not apply a baseline correction,^27^, ^28^, ^33^, ^47^, ^50^ including the study partially overlapping with the current analysis.^38^ Moreover, there is evidence to suggest that this approach may distort poststimulus topographies.^49^, ^50^ However, to enable comparison with analyses in which such corrections have been applied, analyses were rerun with baseline correction (see Supplement II, available online).

### Statistical Analysis

Fourteen preterm-born participants (7.5%) were excluded from the ERP analysis of the Go condition, 16 preterm-born participants (8.6%) were excluded from the ERP analysis of the NoGo condition, and four preterm-born participants (2.2%) were excluded from the ERP analysis of the Cue condition due to having fewer than 20 artifact-free segments available for analysis. Seven participants with ADHD (10.1%) were excluded from the ERP analysis of the Go and NoGo condition, and two participants with ADHD (2.9%) were excluded from the ERP analysis of the Cue condition due to having fewer than 20 artifact-free segments available for analysis. Two control participants (1.5%) were excluded from the ERP analysis of the Go condition, and six control participants (4.4%) were excluded from the ERP analysis of the NoGo condition due to having fewer than 20 artifact-free segments available for analysis.

Data were analyzed using random intercept models in Stata to control for nonindependence of the data (i.e., data coming from siblings of one family), using a “robust cluster” command to estimate standard errors.^51^, ^52^ Regression-based corrections for age were applied to raw scores, and residual scores were analyzed. In addition, we reran all analyses on a carefully age-matched subsample (aged 14−19 years) due to significant group mean differences in age and the possibility of age effects on ERP measures (see Table S2 in Supplement III for descriptive statistics, available online). All analyses controlled for sex. Results are presented both with and without IQ as a covariate to empirically examine the effects of IQ on ERP components. Correlations were also run to examine the associations between ERP measures and DIVA ADHD symptom scores in the preterm group. Effect sizes (Cohen’s *d*), which were calculated using the difference in the means divided by the pooled standard deviation,^53^ are reported. According to Cohen (1988), *d* = 0.20 constitutes a small effect, *d* = 0.50 a medium effect, and *d* = 0.80 a large effect.

## Results

### Cognitive-Performance Measures

No significant main effects of group emerged for MRT (*z* = 1.21, *p* = .225), RTV (*z* = 0.55, *p* = .585), the number of total OE (*z* = −0.38, *p* = .707), and the number of total CE (*z* = −0.27, *p* = .785) (Table 3). Because our previous analysis of these ADHD and control groups showed case−control differences with regard to RTV, we repeated the analysis without the preterm group to see whether we could repeat our previous findings. We suspected that the lack of ADHD−control differences was due to a large preterm group lying intermediate between the ADHD and control groups. The random intercept model yielded significant main effects of group for RTV (*z* = −2.13, *p* = .021), demonstrating significantly lower RTV scores in the ADHD group compared to the control group.

**Table 3:** Cognitive and Event-Related Potential (ERP) Measures From the Cued Continuous Performance Test

	Site	ADHD (n = 69)		Preterm (n = 186)		Control (n = 135)		Cohen’s *d*		
		Mean	SD	Mean	SD	Mean	SD	a	b	c
MRT	—	413.7 (**20.7**)	69.7 (**66.4**)	404.0 (**-2.8**)	71.5 (**69.7**)	385.6 (**-10.1**)	48.0 (**48.0**)	0.34∗	*0.56*∗	0.12∗
RTV	—	109.7 (**23.0**)	59.0 (**57.2**)	97.6 (**-3.0**)	50.9 (**49.0**)	80.2 (**-9.2**)	58.3 (**41.0**)	*0.51*∗	*0.68*∗	0.13∗
OE	—	2.5 (**1.2**)	3.8 (**3.8**)	1.7 (**-0.1**)	4.1 (**4.1**)	0.7 (**-0.7**)	1.4 (**1.4**)	0.32∗	*0.76*∗	0.19∗
CE	—	4.1 (**0.9**)	5.1 (**5.4**)	4.1 (**-0.6**)	9.9 (**9.9**)	1.9 (**-1.6**)	2.2 (**2.3**)	0.17∗	*0.70*∗	0.13∗
Cue-P3	Pz	5.74 (**-0.29**)	3.9 (**3.6**)	7.44 (**-0.27**)	3.9 (**3.8**)	6.37 (**0.22**)	2.6 (**2.4**)	0.01∗	0.18∗	0.15∗
CNV	Cz	-2.30 (**0.89**)	4.7 (**4.7**)	-2.75 (**0.25**)	2.2 (**2.1**)	-3.65 (**-0.50**)	2.0 (**2.0**)	0.21∗	0.44∗	0.36∗
	CPz	-2.31 (**0.78**)	4.5 (**4.5**)	-2.16 (**0.55**)	2.4 (**2.4**)	-3.78 (**-0.79**)	1.8 (**1.8**)	0.07∗	*0.52*∗	*0.62*∗
Go-P3	CPz	8.30 (**0.23**)	6.3 (**6.1**)	8.28 (**-0.76**)	5.2 (**5.1**)	8.81 (**0.54**)	4.1 (**4.0**)	0.18∗	0.07∗	0.28∗
	Pz	9.18 (**0.58**)	6.5 (**6.2**)	8.89 (**-1.17**)	5.2 (**5.0**)	9.93 (**1.05**)	4.3 (**4.1**)	0.33∗	0.10∗	0.48∗
NoGo-P3	FCz	6.14 (**-1.21**)	4.1 (**4.2**)	8.21 (**0.48**)	5.0 (**5.0**)	8.00 (**0.09**)	4.7 (**4.6**)	0.36∗	0.29∗	0.14∗
	Cz	6.77 (**-1.25**)	4.3 (**4.4**)	6.56 (**-0.70**)	4.4 (**4.4**)	9.73 (**1.86**)	4.5 (**4.6**)	0.12∗	0.65∗	0.57∗
NoGo-N2	Fz	-4.95 (**-0.35**)	3.2 (**3.3**)	-6.02 (**0.04**)	4.0 (**3.9**)	-4.69 (**0.20**)	3.2 (**3.1**)	0.10∗	0.17∗	0.04∗

Note: Means, standard deviations (SD), and effect sizes (Cohen’s d) for the attention-deficit/hyperactivity disorder (ADHD), preterm, and control group are shown. Values represent raw scores. Regression-based corrections are shown in bold in parentheses. Moderate effect sizes are shown in italics. a = ADHD vs. Preterm; b = ADHD vs. Control; c = Preterm vs. Control; CE = commission errors; CNV = contingent negative variation; MRT = mean reaction time in milliseconds; OE = omission errors; RTV = reaction time variability in milliseconds.

∗ *p* < .05.

No significant main effect of group emerged for MRT (*z* = 0.40, *p* = .687), RTV (*z* = 0.34, *p* = .733), or the numbers of total omission (OE; *z* = 0.74, *p* = .457) and commission errors (CE; *z* = −0.54, *p* = .588) when IQ was included as a covariate. DIVA ADHD symptom scores in the preterm group were significantly correlated with MRT (*r* = 0.20, *p* = .030), RTV (*r* = 0.22, *p* = .015), OE (*r* = 0.21, *p* = .019), and CE (*r* = 0.22, *p* = .015) when IQ was included as a covariate.

### ERP Results

#### Cue Condition

No significant main effect of group emerged for Cue-P3 amplitude (*z =* −0.35, *p* = .730). Because our previous analysis of the ADHD and control groups showed case−control differences on Cue-P3 amplitude, we repeated the analysis without the preterm group to check whether we could repeat our previous findings. We suspected that the lack of ADHD−control differences was due to a large preterm group lying intermediate between the ADHD and control groups. The random intercept model yielded a significant main effect of group for Cue-P3 amplitude (z = −2.26, *p* = .024), demonstrating significantly reduced Cue-P3 amplitude in the ADHD group compared to the control group. DIVA ADHD symptom scores in the preterm group were not significantly correlated with Cue-P3 amplitude (*r =* 0.018, *p =* .843).

**Figure 1:**
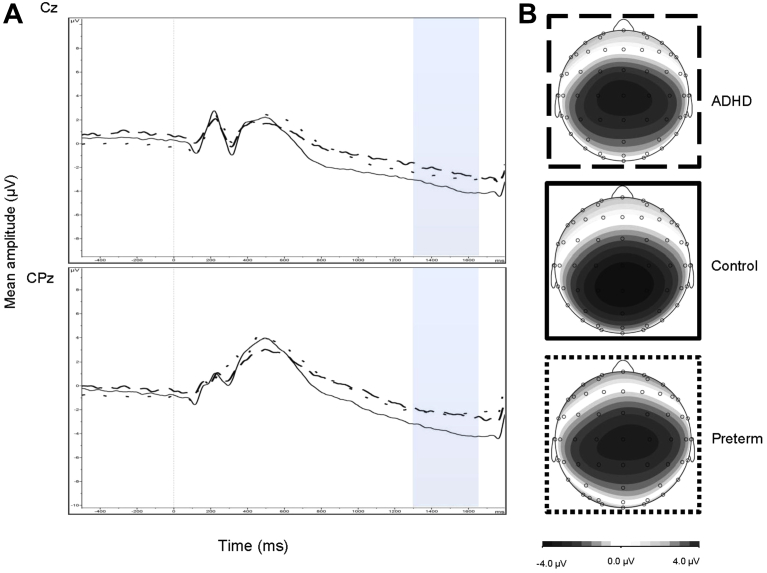
(A) Grand average event-related potentials (ERPs) to cue stimuli at the Cz (above) and CPz (below) electrodes, showing the contingent negative variation (CNV) in the 1,300- to 1,650-millisecond window (attention-deficit/hyperactivity disorder [ADHD] represented by dashed lines; preterm group represented by dotted lines; control group indicated by solid lines), and (B) topographic maps for each group.

The random intercept model yielded a significant main effect of group for CNV amplitude (*z =* 4.15, *p* < .001) and a significant group-by-recording site interaction (*z =* 2.21, *p* = .027) (Figure 1). No significant main effect of recording site (Cz and CPz) (*z =* 0.16, *p* = .875) was found. Post hoc tests revealed that the control group demonstrated significantly greater CNV amplitude at Cz compared to both the ADHD (*t =* 2.54, df = 135, *p =* .012) and preterm groups (*t =* 3.65, df = 215, *p* < .001) with small-to-moderate effect sizes (*d* = 0.44 and *d* = 0.38 respectively) (Table 3). The ADHD and preterm groups did not differ significantly with regard to CNV amplitude at Cz (*t =* −0.25, df = 175, *p =* .801). Further post hoc tests revealed that the control group demonstrated significantly greater CNV amplitude at CPz compared to both the ADHD (*t =* 3.74, df = 135, *p* < .001) and preterm groups (*t =* 6.42, df = 215, *p* < .001), with moderate effect sizes (*d* = 0.60 and *d* = 0.63 respectively) (Table 3). The ADHD and preterm groups did not differ significantly with regard to CNV amplitude at CPz (*t =* 1.02, df = 175, *p =* .311). DIVA ADHD symptom scores in the preterm group were not significantly correlated with CNV amplitude (*r =* 0.018, *p =* .843).

No significant main effect of group emerged for Cue-P3 amplitude (*z =* −1.04, *p =* .299) when IQ was included as a covariate. The random intercept model yielded a significant main effect of group for CNV amplitude (*z =* 4.06, *p* < .001) and a significant group-by-recording site interaction (*z =* 2.21, *p =* .027) when IQ was included as a covariate. No significant main effect of recording site (Cz and CPz) (*z =* 0.16, *p =* .875) was found when IQ was included as a covariate. Post hoc tests revealed that the control group demonstrated significantly greater CNV amplitude at Cz compared to the preterm group (*t =* 2.88, df = 215, *p =* .004) but not compared to the ADHD group (*t =* 1.74, df = 135, *p =* .083). The ADHD and preterm groups did not differ significantly with regard to CNV amplitude at Cz (*t =* 0.19, df = 135, *p =* .849). Further post hoc tests revealed that the control group demonstrated significantly greater CNV amplitude at CPz compared to both the preterm (*t =* 5.21, df = 215, *p* < .001) and ADHD (*t =* 3.27, df = 135, *p* < .001) groups. The ADHD and preterm groups did not differ significantly with regard to CNV amplitude at CPz (*t =* 1.37, df = 175, *p =* .174).

#### Go Condition

**Figure 2:**
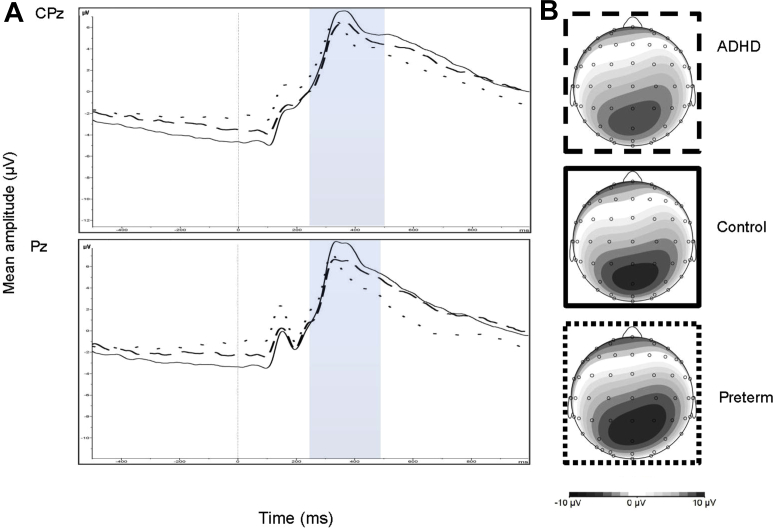
(A) Grand average event-related potentials (ERPs) to Go stimuli at the CPz (above) and Pz (below) electrodes, showing the Go-P3 in the 250- to 500-millisecond window (attention-deficit/hyperactivity disorder [ADHD] represented by dashed lines; preterm group represented by dotted lines; control group indicated by solid lines), and (B) topographic maps for each group.

The random intercept model yielded a significant main effect of group for Go-P3 amplitude (*z =* −2.86, *p =* .004) (Figure 2). No significant main effect of recording site (CPz and Pz) (*z =* 0.14, *p =* .892) and no group-by-recording site interaction (*z =* −1.90, *p =* .057) were found. Post hoc tests revealed that Go-P3 amplitude in the ADHD group was not significantly different from Go-P3 amplitude in the control (*t =* −1.67, df = 131, *p =* .097) and preterm groups (*t =* −1.35, df = 169, *p =* .178). The preterm group demonstrated significantly attenuated Go-P3 amplitude compared to the control group (*t =* −3.08, df = 208, *p =* .002), with a small effect size (*d* = 0.36) (Table 3). DIVA ADHD symptom scores in the preterm group were not significantly correlated with Go-P3 amplitude (*r =* −0.019, *p =* .841).

The random intercept model yielded a significant main effect of group for Go-P3 amplitude (*z =* −2.80, *p =* .005) when IQ was included as a covariate. No significant main effect of recording site (CPz and Pz) (*z =* 0.14, *p =* .892) and no group-by-site interaction (*z =* −1.90, *p =* .058) was found when IQ was included as a covariate. Post hoc tests revealed that Go-P3 amplitude in the ADHD group was not significantly different from Go-P3 amplitude in the control (*t =* −1.63, df = 131, *p =* .105) and preterm groups (*t =* −1.71, df = 169, *p =* .090) when IQ was included as a covariate. The preterm group demonstrated significantly attenuated Go-P3 amplitude compared to the control group (*t =* −2.89, df = 208, *p =* .004) when IQ was included as a covariate.

#### NoGo Condition

**Figure 3:**
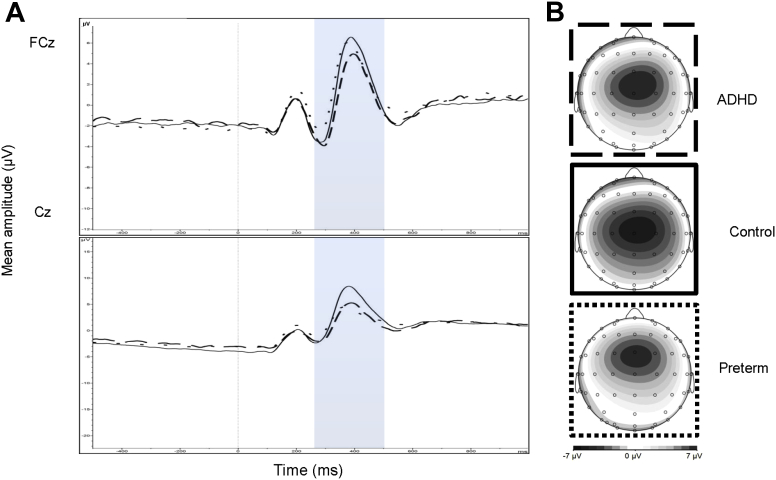
(A) Grand average event-related potentials (ERPs) to NoGo stimuli at the FCz (above) and Cz (below) electrodes, showing the NoGo-P3 in the 250- to 500-millisecond window (attention-deficit/hyperactivity disorder [ADHD] represented by dashed lines; preterm group represented by dotted lines; and the control group indicated by solid lines), and (B) topographic maps for each group.

The random intercept model yielded no significant main effect of group for NoGo-N2 amplitude (*z =* 0.08, *p =* .940). NoGo-N2 amplitude was not previously investigated in this sample of adolescents with ADHD and controls.^38^ For NoGo-P3 amplitude, no significant main effect of group (*z =* −1.01, *p =* .313) and recording site (FCz and Cz) (*z =* 0.03, *p =* .978) emerged (Figure 3). However, a significant group-by-recording site interaction emerged for NoGo-P3 amplitude (*z =* −4.77, *p* < .001). Post hoc tests revealed that the ADHD group showed significantly attenuated NoGo-P3 amplitude at FCz compared to the preterm group (*t =* 2.56, df = 170, *p =* .011), with small effect size (*d* = 0.37), but not compared to the control group (*t =* −1.72, df = 131, *p =* .088). No significant difference in NoGo-P3 amplitude at FCz emerged between preterm and control participants (*t =* 0.41, df = 207, *p =* .685). Although the ADHD and preterm groups demonstrated no significant difference in NoGo-P3 amplitude at Cz (*t =* 0.53, df = 170, *p =* .599), both the ADHD (*t =* −3.84, df = 131, *p* < .001) and preterm (*t =* −4.00, df = 207, *p* < .001) groups showed significantly attenuated NoGo-P3 amplitude at Cz compared to the control group, with moderate effect sizes (*d* = 0.63 and *d* = 0.54). DIVA ADHD symptom scores in the preterm group were significantly negatively correlated with NoGo-P3 amplitude (*r =* −0.20, *p =* .029).

The random intercept model yielded no significant main effect of group for NoGo-N2 amplitude (*z =* −0.20, *p =* .838) when IQ was included as a covariate. For NoGo-P3 amplitude, no significant main effect of group (*z =* −0.84, *p =* .403) and recording site (FCz and Cz) (*z =* 0.03, *p =* .978) emerged when IQ was included as a covariate. However, a significant group-by-recording site interaction emerged for NoGo-P3 amplitude (*z =* −4.79, *p* < .001) when IQ was included as a covariate. Post hoc tests revealed that the ADHD group showed significantly attenuated NoGo-P3 amplitude at FCz compared to the preterm group (*t =* 2.01, df = 170, *p =* .046), but not compared to the control group (*t =* −1.38, df = 131, *p =* .170). No significant difference in NoGo-P3 amplitude at FCz emerged between preterm and control participants (*t =* 0.67, df = 207, *p =* .504). Although the ADHD and preterm groups demonstrated no significant difference in NoGo-P3 amplitude at Cz (*t =* 0.08, df = 170, *p =* .938), both the ADHD (*t =* −3.29, df = 131, *p* < .001) and preterm groups (*t =* −3.53, df = 207, *p* < .001) showed significantly attenuated NoGo-P3 amplitude at Cz compared to the control group when IQ was included as a covariate.

Excluding the eight preterm-born individuals meeting diagnostic criteria for a research diagnosis of ADHD from the analyses did not alter the results (Figures S1−S3, available online).

With the exception of the CNV findings, cognitive-performance and ERP results remain unchanged in the age-matched analysis (Table S3, available online). The preterm group of the age-matched subsample demonstrated significantly attenuated CNV amplitude compared to the control group but not compared to the ADHD group. The ADHD and control groups of the age-matched sample did not differ significantly with regard to CNV amplitude.

## Discussion

Here, we directly compared preterm-born adolescents to term-born adolescents with ADHD and term-born controls on cognitive performance and ERP measures from the CPT-OX, which sensitively capture the attentional and inhibitory impairments in adolescents with ADHD. We provide evidence for executive response control (Go-P3), response preparation (CNV), and response inhibition (NoGo-P3) impairments in preterm-born individuals. Although the observed impairment in executive response control (Go-P3) represents preterm birth-specific impairments, response preparation (CNV) and response inhibition (NoGo-P3) impairments were also observed in individuals with ADHD.^38^ The current study constitutes the first cognitive-neurophysiological comparison of attentional and inhibitory processing in adolescents born preterm and term-born adolescents with ADHD using a detailed measure of attention and inhibitory control, and furthers our understanding of impairments of response preparation, executive response control, and response inhibition in preterm-born adolescents.

Our ERP results show a significant group difference in P3 amplitude in response to Go stimuli, which was reduced in preterm-born adolescents compared to the control group. The Go-P3 has been linked to several attentional functions, such as evaluation of stimuli and resource allocation,^54^, ^55^ and may reflect aspects of executive response control.^27^, ^28^ Although an association between preterm birth and lower mean IQ scores has consistently been reported,^56^ our finding of a preterm birth−specific impairment is unlikely due to lower general cognitive ability, because our preterm group did not differ significantly from the control group on IQ scores, and including IQ as a covariate had no effect on the results. However, as this is the first investigation of its kind, the finding of reduced Go-P3 amplitude in preterm-born adolescents compared to controls requires replication. In line with the majority of previous research, we found no Go-P3 differences between the ADHD and control groups.^27^, ^28^, ^35^, ^57^

Response inhibition impairments, as indexed by abnormal NoGo-P3 amplitude, were observed in the preterm-born adolescents. These impairments were linked to the increased ADHD symptoms in the preterm group. Our previous research had also established response inhibition impairments, reflected by decreased NoGo-P3 amplitude, in adolescents with ADHD.^38^ The significant group-by-recording site interactions in the current study, supported by topographic maps of NoGo-P3 mean amplitude, indicate, however, a degree of group specificity in relation to response inhibition impairments. Although both the ADHD^38^ and preterm groups demonstrated significantly reduced NoGo-P3 amplitude compared to controls at the vertex, the preterm group showed a more frontal scalp distribution of the NoGo-P3 component compared to adolescents with ADHD and controls, which was significantly greater than NoGo-P3 amplitude in the ADHD group, suggesting greater neurophysiological immaturity.^29^, ^58^, ^59^ These neurophysiological findings extend previous cognitive research, which has indicated impairments in response inhibition in individuals born preterm,^60^, ^61^, ^62^ and suggest an overlapping neurophysiological impairment as well as a degree of group specificity.

We further identified the same CNV abnormalities in the preterm group as observed in individuals with ADHD,^38^ suggestive of impaired attentional orienting and response preparation.^57^ The attenuated CNV amplitude in adolescents born preterm is in accordance with previous evidence of abnormalities in attentional orienting as indexed by larger N2 and reduced P3a components in children born preterm.^24^, ^25^, ^26^ The lack of a difference among the three groups with regard to conflict monitoring as indexed by NoGo-N2 amplitude is inconsistent with previous research demonstrating abnormalities in NoGo-N2 amplitude in children born very preterm (<32 weeks) using an auditory oddball paradigm,^24^, ^26^ as well as in individuals with ADHD using flanker tasks.^63^, ^64^, ^65^ However, the CPT-OX is a task with relatively low conflict-monitoring demands, and previous studies using the CPT-OX have also not been able to demonstrate an attenuated NoGo-N2 in individuals with ADHD.^27^, ^28^, ^47^ Similarly, we found no difference in Cue-P3 amplitude between the preterm-born adolescents and the ADHD and control groups. These findings do not replicate auditory ERP studies, which demonstrated abnormalities in early attentional ERP components (P1 and P3a) in preterm-born children.^24^, ^25^, ^26^ However, overall research is limited, and no study to date has investigated attention orienting as indexed by Cue-P3 amplitude in adolescents born preterm. Further studies should explore the differences between individuals born preterm and individuals with ADHD in tasks with higher conflict-monitoring demands to examine conflict monitoring as indexed by NoGo-N2 amplitude. In addition, future research needs to establish whether individuals born preterm demonstrate impairments in attentional orienting as indexed by Cue-P3 amplitude.

No significant differences in mean reaction time (MRT), reaction time variability (RTV), omission errors (OE), and commission errors (CE) were found between the preterm and control groups, suggesting that preterm-born adolescents may be able to compensate for the impairment seen in the brain processes involved in response preparation, executive response control, and response inhibition. As reported previously, the ADHD group demonstrated significantly increased MRT and RTV and a greater number of OEs compared to both preterm and control groups,^38^ in line with previous research.^8^, ^10^, ^11^, ^12^, ^66^

A limitation of this study is that we were unable to investigate whether risk factors for being preterm (e.g., poverty, malnutrition) might account for the ERP findings in our sample. Although population-based quasi-experimental designs have established that the associations between preterm birth and psychiatric morbidity are largely independent of shared familial confounds and measured covariates, consistent with a causal inference,^5^ future research should take risk factors for preterm birth into account when examining cognitive-neurophysiological measures in preterm-born individuals. Finally, the DIVA is a diagnostic interview for adult ADHD, but was used across ages in this study for consistency. The DIVA follows *DSM* criteria for ADHD closely. It inquires about the presence of ADHD symptoms in adulthood as well as in childhood, and about the chronicity of these symptoms, and it can therefore be easily adapted for use in younger participants. Nonetheless, future studies should apply diagnostic measures normed for their population.

In conclusion, this study provides evidence for impairments in brain processes involved in response preparation, executive response control, and response inhibition in adolescents born preterm. Although some of the impairments found in adolescents born preterm overlap with those found in term-born adolescents with ADHD, the preterm group also shows unique impairments, indicating more wide-ranging impairments in the preterm group, compared to individuals with ADHD. This idea is supported by research suggesting that, as well as being a risk factor for ADHD, preterm birth presents a risk factor for other psychiatric disorders, such as schizophrenia, bipolar disorder, and autism spectrum disorder.^5^, ^67^, ^68^, ^69^ The late third trimester (32–40 weeks’ gestation) serves as a critical period to lay the foundation of vital brain networks.^70^, ^71^ It is therefore conceivable that preterm birth may result in trauma to the brain networks associated with ADHD, as well as networks associated with additional impairments. These results, and the findings that ADHD symptom scores were increased in the preterm group compared to controls, suggest that preterm birth may present a risk factor for both ADHD and additional impairments. Routine psychiatric screening to facilitate early psychological referral is therefore likely to be beneficial in this vulnerable population. As this is one of the first studies to directly compare preterm-born adolescents to term-born adolescents with ADHD and term-born controls on cognitive performance and ERP measures of attention and inhibitory control, these findings require replication in larger-scale studies with similar-size groups and additional research looking at broader forms of psychopathology.
