# Corrigenda

**DOI:** 10.1016/j.jaac.2017.11.007

**Published:** 2018-01

In the research article “Association of Preterm Birth With Attention-Deficit/Hyperactivity Disorder–Like and Wider-Ranging Neurophysiological Impairments of Attention and Inhibition” by Anna-Sophie Rommel, Sarah-Naomi James, Gráinne McLoughlin, Daniel Brandeis, Tobias Banaschewski, Philip Asherson, and Jonna Kuntsi, published in the January 2017 issue of the *Journal* (2017;56:40-50), an error appeared in the Discussion section, which stated that there were no significant differences in regard to IQ between the preterm and control groups. The sentence as published reads: “Although an association between preterm birth and lower mean IQ scores has consistently been reported,^56^ our finding of a preterm birth–specific impairment is unlikely due to lower general cognitive ability, because our preterm group did not differ significantly from the control group on IQ scores, and including IQ as a covariate had no effect on the results.” The sentence should instead read: “Although an association between preterm birth and lower mean IQ scores has consistently been reported,^56^ our finding of a preterm birth–specific impairment is unlikely due to lower general cognitive ability, because including IQ as a covariate had no effect on the results.”

In addition, in Table 3 on page 44, all group comparisons are currently incorrectly indicated as significant by asterisks. A revised table can be found below. The authors regret the error.

**Table 3:** Cognitive and Event-Related Potential (ERP) Measures From the Cued Continuous Performance Test

	Site	ADHD (n = 69)		Preterm (n = 186)		Control (n = 135)		Cohen’s *d*		
		Mean	SD	Mean	SD	Mean	SD	a	b	c
MRT	—	413.7 (**20.7**)	69.7 (**66.4**)	404.0 (**-2.8**)	71.5 (**69.7**)	385.6 (**-10.1**)	48.0 (**48.0**)	0.34	*0.56*	0.12
RTV	—	109.7 (**23.0**)	59.0 (**57.2**)	97.6 (**-3.0**)	50.9 (**49.0**)	80.2 (**-9.2**)	58.3 (**41.0**)	*0.51*	*0.68*	0.13
OE	—	2.5 (**1.2**)	3.8 (**3.8**)	1.7 (**-0.1**)	4.1 (**4.1**)	0.7 (**-0.7**)	1.4 (**1.4**)	0.32	*0.76*	0.19
CE	—	4.1 (**0.9**)	5.1 (**5.4**)	4.1 (**-0.6**)	9.9 (**9.9**)	1.9 (**-1.6**)	2.2 (**2.3**)	0.17	*0.70*	0.13
Cue-P3	Pz	5.74 (**-0.29**)	3.9 (**3.6**)	7.44 (**-0.27**)	3.9 (**3.8**)	6.37 (**0.22**)	2.6 (**2.4**)	0.01	0.18	0.15
CNV	Cz	-2.30 (**0.89**)	4.7 (**4.7**)	-2.75 (**0.25**)	2.2 (**2.1**)	-3.65 (**-0.50**)	2.0 (**2.0**)	0.21	0.44∗	0.36∗
	CPz	-2.31 (**0.78**)	4.5 (**4.5**)	-2.16 (**0.55**)	2.4 (**2.4**)	-3.78 (**-0.79**)	1.8 (**1.8**)	0.07	*0.52*∗	*0.62*∗
Go-P3	CPz	8.30 (**0.23**)	6.3 (**6.1**)	8.28 (**-0.76**)	5.2 (**5.1**)	8.81 (**0.54**)	4.1 (**4.0**)	0.18	0.07	0.28∗
	Pz	9.18 (**0.58**)	6.5 (**6.2**)	8.89 (**-1.17**)	5.2 (**5.0**)	9.93 (**1.05**)	4.3 (**4.1**)	0.33∗	0.10	0.48∗
NoGo-P3	FCz	6.14 (**-1.21**)	4.1 (**4.2**)	8.21 (**0.48**)	5.0 (**5.0**)	8.00 (**0.09**)	4.7 (**4.6**)	0.36∗	0.29	0.14
	Cz	6.77 (**-1.25**)	4.3 (**4.4**)	6.56 (**-0.70**)	4.4 (**4.4**)	9.73 (**1.86**)	4.5 (**4.6**)	0.12	0.65∗	0.57∗
NoGo-N2	Fz	-4.95 (**-0.35**)	3.2 (**3.3**)	-6.02 (**0.04**)	4.0 (**3.9**)	-4.69 (**0.20**)	3.2 (**3.1**)	0.10	0.17	0.04

Note: Values represent raw scores. Regression-based corrections are shown in bold in parentheses. Moderate effect sizes are shown in italics. a = attention-deficit/hyperactivity disorder (ADHD) vs. Preterm; b = ADHD vs. Control; c = Preterm vs. Control; CE = commission errors; CNV = contingent negative variation; MRT = mean reaction time in milliseconds; OE = omission errors; RTV = reaction time variability in milliseconds.

∗ *p* < .05.

