# Supplementary material for: Association of Preterm Birth With Attention-Deficit/Hyperactivity Disorder–Like and Wider-Ranging Neurophysiological Impairments of Attention and Inhibition
Source: J Am Acad Child Adolesc Psychiatry. 2017 Jan;56(1):40–50. doi: 10.1016/j.jaac.2016.10.006 (PMC5196005; doi:10.1016/j.jaac.2016.10.006)
Supplement: Supplemental Material [file mmc1.docx]

## Supplement I – Results Without the Preterm-Born Individuals With a Research Diagnosis of Attention-Deficit/Hyperactivity Disorder (ADHD)

The subsample consisted of 69 participants with ADHD, 178 preterm-born participants, and 135 controls. Significant differences in gestational age (GA), IQ, age, and gender distribution were observed (Table S1). The ADHD group showed significantly higher Conners’ parent-rated ADHD symptoms and functional impairment scores on the Barkley Functional Impairment Scale than both the preterm (t=-17.65, df=105, p<.001 and t=-19.30, df=105, p<.001, respectively) and control groups (t=19.38, df=79, p<.001 and t= 17.13, df=79, p<.001, respectively). The preterm group further demonstrated significantly higher Conners’ parent-rated ADHD symptom scores (t=2.63, df=136, p=.009), but not functional impairment scores on the Barkley Functional Impairment Scale (t= 0.02, df=136, p= .985), compared to the control group.

#### **Cognitive-Performance Measures**

No significant main effects of group emerged for mean reaction time (MRT) (z=1.01, p=.314), reaction time variability (RTV) (z=0.30, p=.763), the number of total omission errors (OE) (z=0.30, p=.766), and the number of total commission errors (CE) (z=-0.41, p=.679). The Diagnostic Interview for ADHD in adults (DIVA) ADHD symptom scores in the preterm group were not significantly correlated with MRT (r=0.17, p=.061), but were significantly and positively correlated with RTV (r= 0.22, p= .019), OE (r=0.24, p=.007), and CE (r=0.27, p=.003).

#### **ERP Results**

##### *Cue Condition*

No significant main effect of group emerged for Cue-P3 amplitude (z=-0.35, p=.726). The random intercept model yielded a significant main effect of group for contingent negative variation (CNV) amplitude (z=3.87, p<.001) (Figure 1). No significant main effect of recording site (Cz and CPz) (z=0.16, p=0.875) and no significant group-by-recording site interaction (z=0.12, p=.901) were found. Post hoc tests revealed that the control group demonstrated significantly greater CNV amplitude at Cz compared to both the ADHD (t=2.54, df=135, p=.012) and preterm groups (t=3.16, df=209, p=.002) with small-to-moderate effect sizes (d=0.44 and d=0.48, respectively). The ADHD and preterm groups did not differ significantly with regard to CNV amplitude at Cz (t=-0.30, df=175, p=.765). Further post hoc tests revealed that the control group demonstrated significantly greater CNV amplitude at CPz compared to both the ADHD (t=3.74, df=135, p<.001) and preterm groups (t=5.45, df=215, p<.001), with moderate effect sizes (d=0.60 and d=0.63, respectively). The ADHD and preterm groups did not differ significantly with regard to CNV amplitude at CPz (t=1.02, df=175, p=.311). DIVA ADHD symptom scores in the preterm group were not significantly correlated with Cue-P3 (r=0.016, p=.860) and CNV (r=0.008, p=.892) amplitude.

##### *Go Condition*

The random intercept model yielded a significant main effect of group for Go-P3 amplitude (z=-2.72, p=.007). No significant main effect of recording site (CPz and Pz) (z=0.14, p=.892) and no group-by-recording site interaction (z=-1.90, p=.057) were found. Post hoc tests revealed that Go-P3 amplitude in the ADHD group was not significantly different from Go-P3 amplitude in the control (t=-1.67, df=131, p=.097) and preterm groups (t=-1.46, df=167, p=.147). The preterm group demonstrated significantly attenuated Go-P3 amplitude compared to the control group (t=-2.99, df=203, p=.003), with a small effect size (d=0.36). DIVA ADHD symptom scores in the preterm group were not significantly correlated with Go-P3 amplitude (r=-0.0008, p=.990).

##### *NoGo Condition*

The random intercept model yielded no significant main effect of group for NoGo-N2 amplitude (z=0.05, p=.960). NoGo-N2 amplitude was not previously investigated in this sample of ADHD and control adolescents.^1^ For NoGo-P3 amplitude, no significant main effect of group (z=-0.92, p=.357) and recording site (FCz and Cz) (z=0.06, p=.950) emerged (Figure 3). However, a significant group-by-recording site interaction emerged for NoGo-P3 amplitude (z=-4.86, p<.001). Post hoc tests revealed that the ADHD group showed significantly attenuated NoGo-P3 amplitude at FCz compared to the preterm group (t=2.47, df=167, p=.015), with small effect size (d=0.37), but not compared to the control group (t=-1.72, df=131, p=.088). No significant difference in NoGo-P3 amplitude at FCz emerged between preterm and control participants (t=0.37, df=203, p=.714). While the ADHD and preterm groups demonstrated no significant difference in NoGo-P3 amplitude at Cz (t=0.39, df=167, p=.695), both the ADHD (t=-3.84, df=131, p<.001) and preterm groups (t=-4.03, df=203, p<.001) showed significantly attenuated NoGo-P3 amplitude at Cz compared to the control group, with moderate effect sizes (d=0.63 and d=0.54). DIVA ADHD symptom scores in the preterm group were not significantly correlated with NoGo-P3 amplitude (r=-0.14, p=.111).

**Supplement II – Baseline-Corrected Event-Related Potential (ERP) Results**

**Cue Condition**

No significant main effect of group emerged for baseline-corrected Cue-P3 amplitude (z=-0.89, p=.375). The random intercept model yielded a significant main effect of group for baseline-corrected CNV amplitude (z=5.76, p<.001). No significant main effect of recording site (Cz and CPz) (z=0.00, p=1.00) and no significant group-by-recording site interaction (z=0.0, p=1.00) were found. Post hoc tests revealed that the control group demonstrated significantly greater baseline-corrected CNV amplitude compared to the ADHD (t=2.89, df=137, p=.005) and preterm groups (t=4.97, df=215, p<.001). The ADHD and preterm groups did not differ significantly with regard to baseline-corrected CNV amplitude (t=1.29, df=175, p=.199).

**Go Condition**

The random intercept model yielded a significant main effect of group for baseline-corrected Go-P3 amplitude (z=-4.54, p=.004). No significant main effect of recording site (CPz and Pz) (z=0.15, p=.884) and no group-by-site interaction (z=-1.14, p=.255) was found. Post hoc tests revealed that baseline-corrected Go-P3 amplitude in the ADHD group was not significantly different from baseline-corrected Go-P3 amplitude in the preterm group (t=-1.84, df=169, p=.067). The control group demonstrated significantly greater baseline-corrected Go-P3 amplitude compared to both the preterm (t=-4.73, df=208, p<.001) and ADHD groups (t=-3.15, df=131, p=.002).

**NoGo Condition**

The random intercept model yielded no significant main effect of group for baseline-corrected NoGo-N2 amplitude (z=1.26, p=.280). For baseline-corrected NoGo-P3 amplitude, no significant main effect of group (z=-1.03, p=.304) and recording site (FCz and Cz) (z=0.02, p=.981) emerged. However, a significant group-by-recording site interaction emerged for baseline-corrected NoGo-P3 amplitude (z=-4.73, p<.001). Post hoc tests revealed that the ADHD group showed significantly attenuated baseline-corrected NoGo-P3 amplitude at FCz compared to the preterm group (t=2.52, df=170, p=.013) and at trend-level attenuated NoGo-P3 amplitude at FCz compared to the control group (t=-1.91, df=131, p=.058). No significant difference in baseline-corrected NoGo-P3 amplitude at FCz emerged between preterm and control participants (t=0.66, df=207, p=.510). While the ADHD and preterm groups demonstrated no significant difference in NoGo-P3 amplitude at Cz (t=0.71, df=170, p=.476), both the ADHD (t=-4.31, df=131, p<.001) and preterm groups (t=-4.22, df=207, p<.001) showed significantly attenuated baseline-corrected NoGo-P3 amplitude at Cz compared to the control group.

**Supplement III – Analysis of an Age-Matched Subsample**

The final age-matched subsample consisted of 31 participants with ADHD (five sibling pairs and 26 singletons), 97 preterm-born participants (13 sibling pairs and 68 singletons) and 63 controls (eight sibling pairs and 47 singletons). The groups did not differ in terms of age or IQ (Table S1). Significant differences in gender distribution and GA were observed. The ADHD group showed significantly higher functional impairment scores on the Barkley Functional Impairment Scale than both the preterm (t=-10.26, df=105, p<.001) and control groups (t=12.00, df=79, p<.001). The preterm group further demonstrated significantly higher functional impairment scores on the Barkley Functional Impairment Scale than the control group (t=2.67, df=136, p=.008).

#### **Cognitive-Performance Measures**

No significant main effects of group emerged for mean reaction time (MRT) (z=0.63, p=.529), reaction time variability (RTV) (z=0.12, p=.905), the total number of omission errors (OE) (z=-0.21, p=.835), or the total number of commission errors (CE) (z=-0.22, p=.825).

##### **Cue Condition**

No significant main effect of group emerged for Cue-P3 amplitude (z=-0.11, p=.914). The random intercept model yielded a significant main effect of group for CNV amplitude (z=4.34, p<.001) and a significant group-by-recording site interaction (z=2.70, p=.007) (Figure S1). No significant main effect of recording site (Cz and CPz) (z=-0.01, p=.995) was found. Post hoc tests revealed that the preterm group demonstrated significantly attenuated CNV amplitude compared to the control group (t=-2.62, df=138, p=.010), with moderate effect size (d=0.49) (Table S2), but not compared to the ADHD group (t=1.06, df=115, p=.291). The ADHD and control groups did not differ significantly with regard to CNV amplitude (t=0.66, df=87, p=.511).

##### **Go Condition**

A significant main effect of group emerged for Go-P3 amplitude (z=-2.84, p=.005) (Figure S2). Post hoc tests revealed significantly reduced Go-P3 amplitude in the preterm group compared to the control (t=-2.53, df=144, p=.013) and ADHD groups (t=-2.46, df=115, p=.016), with moderate (d=0.57, d=0.46, respectively) effect sizes (Table S2). The ADHD and control groups did not differ with regard to Go-P3 amplitude (t=-0.25, df=89, p=.802).

##### **NoGo Condition**

No significant main effect of group emerged for NoGo-N2 amplitude (z=-0.09, p=.927). No significant main effect of group (z=-0.76, p=.447) or recording site (FCz and Cz) (z=0.10, p=.924) emerged for NoGo-P3 amplitude. However, a significant group-by-recording site interaction emerged for NoGo-P3 amplitude (z=5.80, p<.001) (Figure S3). Post hoc tests revealed that the preterm group showed significantly increased NoGo-P3 amplitude compared to the ADHD group at FCz (t=-2.85, df=107, p=.005), with moderate effect size (d=0.59) (Table S2), but not at Cz (t=0.30, df=108, p=.763). In addition, the preterm group demonstrated significantly reduced NoGo-P3 amplitude compared to the control group at Cz (t=3.53, df=147, p<.001), with moderate effect size (d=0.59), but the two groups did not differ significantly at FCz (t=-1.74, df=130, p=.084). The ADHD group demonstrated significantly reduced NoGo-P3 amplitude compared to the control group at Cz (t=2.34, df=130, p=.001), with moderate effect size (d=0.44), but not at FCz (t=1.33, df=90, p=.187).

Table S1. Descriptive Statistics for the Sample Without the Preterm-Born Individuals With a Research Diagnosis of Attention-Deficit/Hyperactivity Disorder (ADHD)

|  | **ADHD** | **Preterm** | **Control** | **Statistic** | **p-value** |
| --- | --- | --- | --- | --- | --- |
|  | n=69 | n=178 | n=135 | - | - |
| **GA in weeks (SD)** | 39.9 (1.4) | 33.0 (2.9) | 39.9 (1.3) | t=-22.1 | <.001 |
| **IQ (SD)** | 97.7 (13.8) | 105.1 (12.3) | 110.4 (12.2) | t=-2.9 | .004 |
| **Age (SD)** | 18.5 (3.0) | 15.1 (1.8) | 17.8 (2.1) | t=-11.1 | <.001 |
| **Age range** | 12.7-25.9 | 12.0-20.0 | 11.9-21.6 | - | - |
| **Males %** | 88.4 | 54.0 | 75.6 | t=4.6 | <.001 |
| **Conners parent-rated ADHD symptom score (SD)** | 35.8 (10.6) | 10.7 (8.7) | 7.0 (5.6) | t=-1.1 | .294 |
| **BFIS score (SD)** | 16.4 (5.4) | 3.5 (3.7) | 2.1 (2.5) | t=-0.98 | .329 |

Note: BFIS= Barkley Functional Impairment Scale; GA = gestational age.

**Table S2. Descriptive Statistics for the Age-Matched Subsample**

|  | **ADHD** | **Preterm** | **Control** | **Statistic** | **p-value** |
| --- | --- | --- | --- | --- | --- |
| ***Age-matched subsample*** | n=36 | n=94 | n=63 | - | - |
| **GA in weeks (SD)** | 39.72 (1.3) | 33.5 (2.4) | 40.0 (1.10) | z=-18.0 | <.001 |
| **IQ (SD)** | 96.4 (14.0) | 103.6 (13.2) | 108.0 (12.4) | z=-1.6 | .112 |
| **Age (SD)** | 16.6 (1.5) | 16.4 (1.1) | 16.7 (1.0) | z=-1.7 | .082 |
| **Age range** | 14.1-18.9 | 14.8-18.8 | 14.4-18.1 | - | - |
| **Males %** | 88.9 | 44.7 | 47.5 | χ^2^=24.1 | <0.001 |

Note: ADHD = attention-deficit/hyperactivity disorder; GA = gestational age.

| Table S3. Cognitive and Event-Related Potential Measures From the Cued Continuous Performance Test | | | | | | | | | | |
| --- | --- | --- | --- | --- | --- | --- | --- | --- | --- | --- |
| ***Age-matched***  ***subsample*** | **Site** | **ADHD**  (n=36) | | **Preterm**  (n=94) | | **Control**  (n=63) | | **Cohen’s d** | | |
|  |  | Mean | SD | Mean | SD | Mean | SD | **a** | **b** | **c** |
| **MRT** | **-** | 412.50 | 51.5 | 391.44 | 70.7 | 386.44 | 43.9 | 0.32* | *0.56** | 0.08* |
| **RTV** | **-** | 110.53 | 54.3 | 86.51 | 47.2 | 84.40 | 38.8 | 0.48* | *0.58** | 0.05* |
| **OE** | - | 2.58 | 4.2 | 0.80 | 1.5 | 0.81 | 1.9 | *0.66** | *0.60** | 0.00* |
| **CE** | - | 1.50 | 2.0 | 0.84 | 1.7 | 0.98 | 1.4 | 0.36* | 0.31* | 0.09* |
| **Cue-P3** | Pz | 5.50 | 3.7 | 5.88 | 3.0 | 6.55 | 2.5 | 0.12* | 0.36* | 0.24* |
| **CNV** | Cz | -3.23 | 1.5 | -2.82 | 2.0 | -3.77 | 1.9 | 0.25* | 0.28* | 0.49* |
| **Go-P3** | Pz | 9.90 | 3.6 | 7.63 | 5.0 | 10.36 | 3.8 | 0.46* | 0.12* | *0.57** |
| **NoGo-P3** | FCz | 6.26 | 4.1 | 8.87 | 5.9 | 7.55 | 5.3 | *0.59** | 0.29* | 0.28* |
|  | Cz | 7.49 | 4.9 | 7.05 | 5.3 | 9.44 | 4.1 | 0.10* | 0.44* | *0.59** |
| **NoGo-N2** | Fz | -5.44 | 3.3 | -5.04 | 3.6 | -4.78 | 3.2 | 0.11* | 0.24* | 0.11* |
| Note: Means, standard deviations (SD), and effect sizes (Cohen’s d) for the attention-deficit/hyperactivity disorder (ADHD), preterm, and control groups of the age-matched subsample are shown. Values represent raw scores. Moderate effect sizes are shown in italics. a=ADHD vs. Preterm; b=ADHD vs. Control; c=Preterm vs. Control; CE=commission errors; CNV=contingent negative variation; MRT=mean reaction time in ms; OE=omission errors; RTV=reaction time variability in ms.  *p<.05 | | | | | | | | | | |

Mean amplitude (μV)


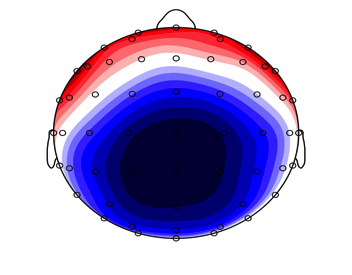

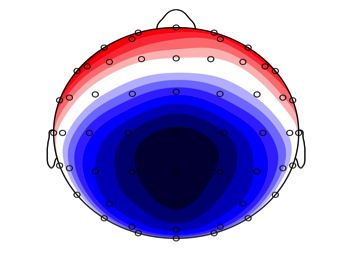

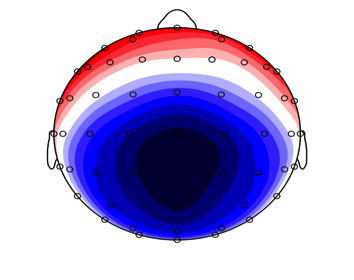


ADHD

Control

Preterm

**A**


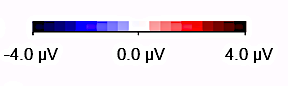


0.0 μV

**B**

Time (ms)


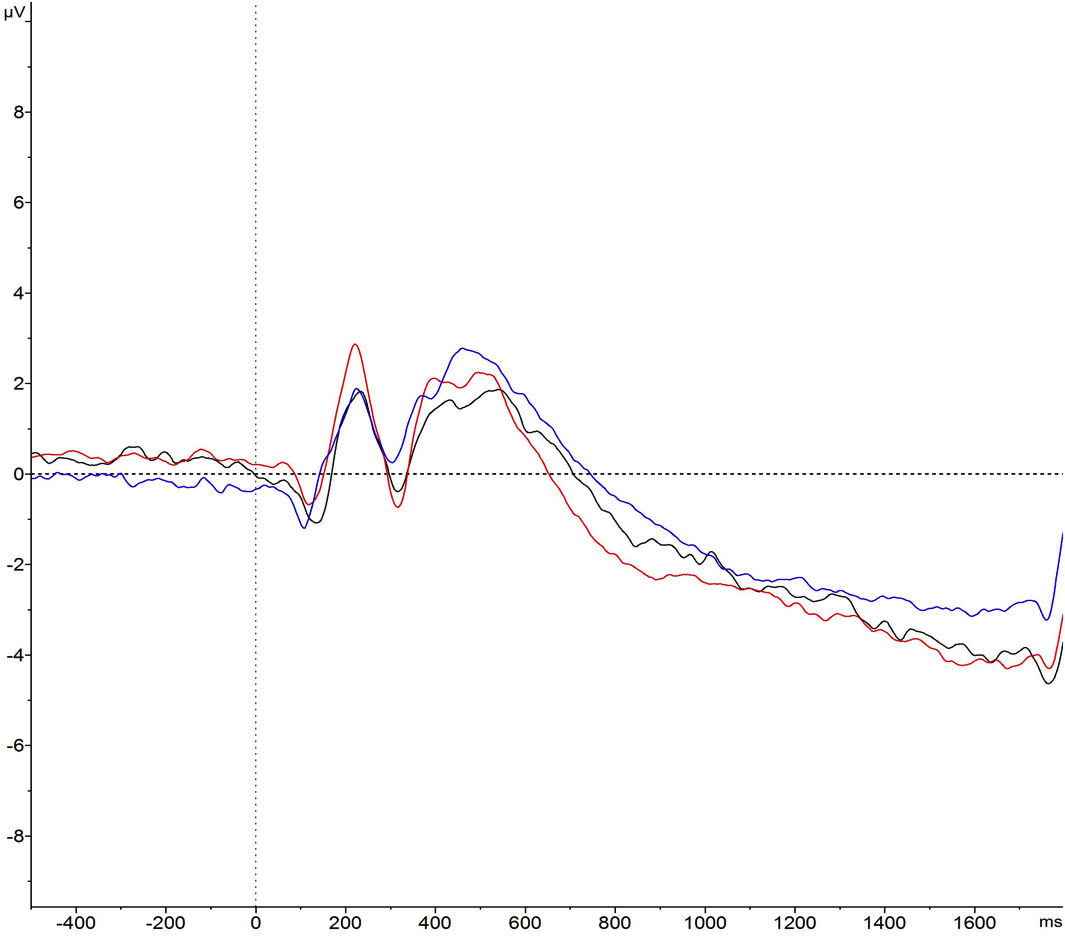


4.0 μV

-4.0 μV

Mean amplitude (μV)

Cz

**Figure S1.** (**A**) Grand average event-related potentials (ERPs) to cue stimuli at the Cz electrode in the age-matched subsample, showing the contingent negative variation (CNV) in the 1300-1650 ms window (attention-deficit/hyperactivity disorder [ADHD] shown in black; the preterm group shown in blue; and the control group shown in red), and (**B**) topographic maps for each group.


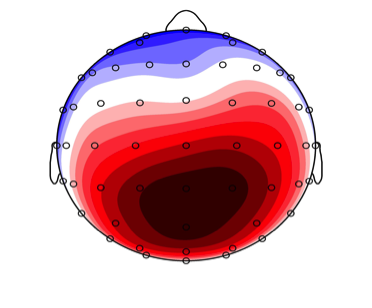

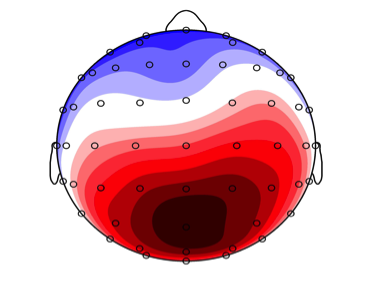

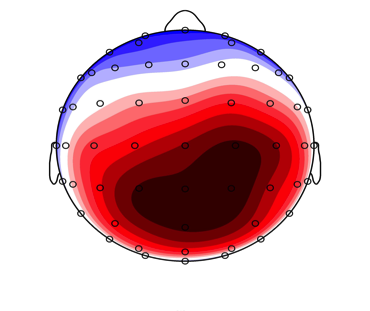


ADHD

Preterm

Control

Mean amplitude (μV)

Time (ms)

**A**

**B**


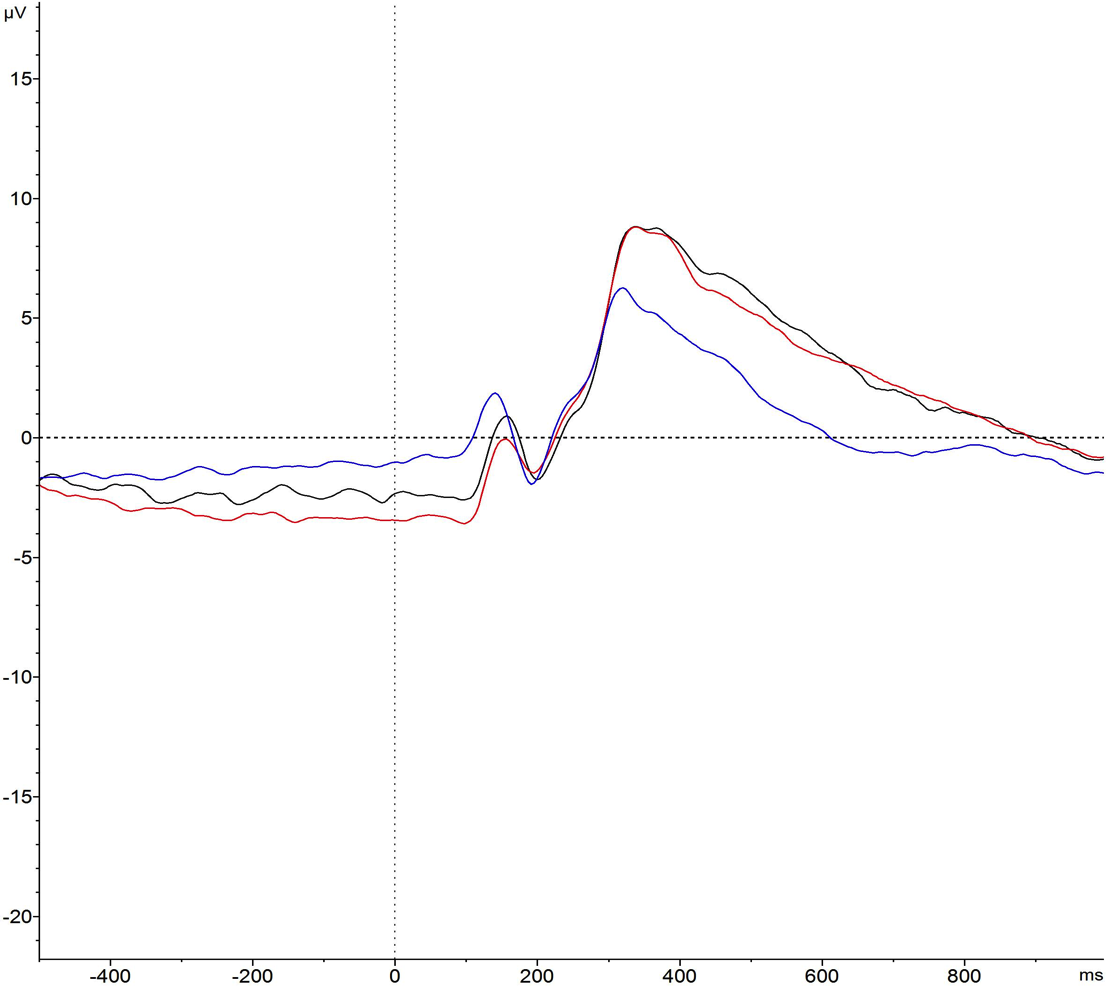


Pz


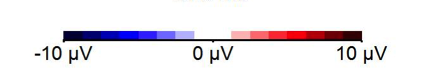


**Figure S2.** (**A**) Grand average event-related potentials (ERPs) to Go stimuli at the Pz electrode in the age-matched subsample, showing the Go-P3 in the 250-500 ms window (attention-deficit/hyperactivity disorder [ADHD] shown in black; the preterm group shown in blue; and the control group shown in red), and (**B**) topographic maps for each group.

**B**

ADHD

Control

Preterm


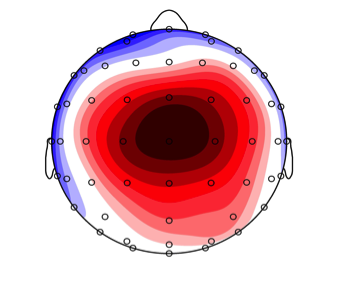

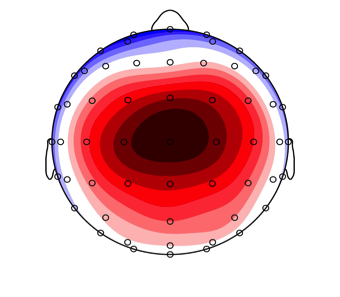

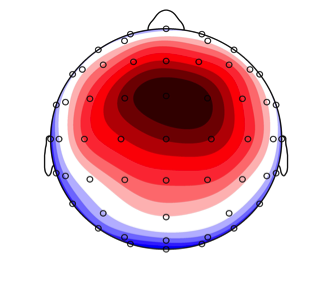

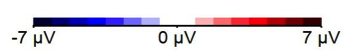


**A**

Time (ms)

Mean amplitude (μV)


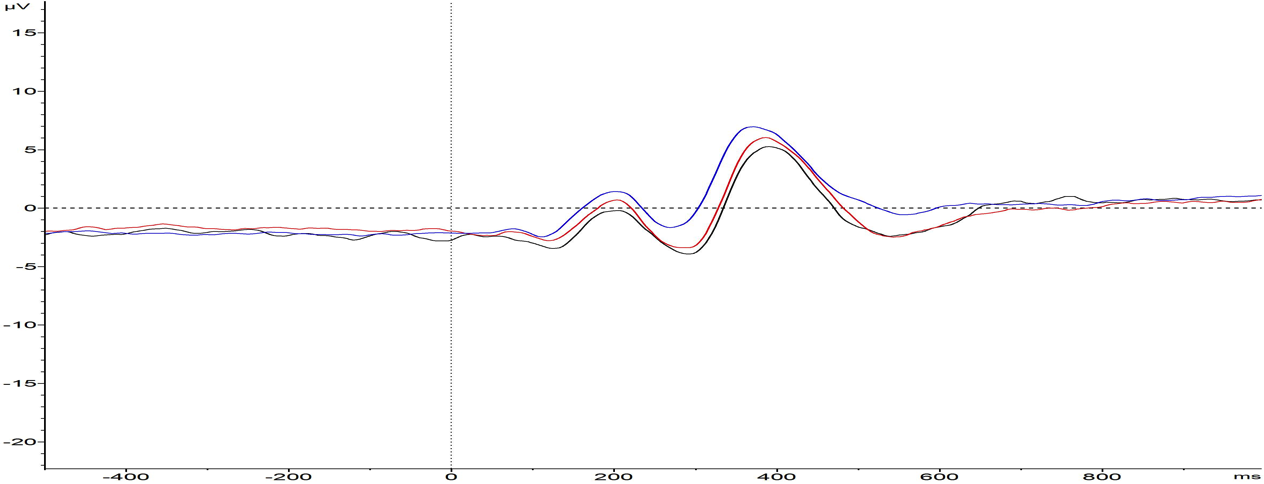


FCz


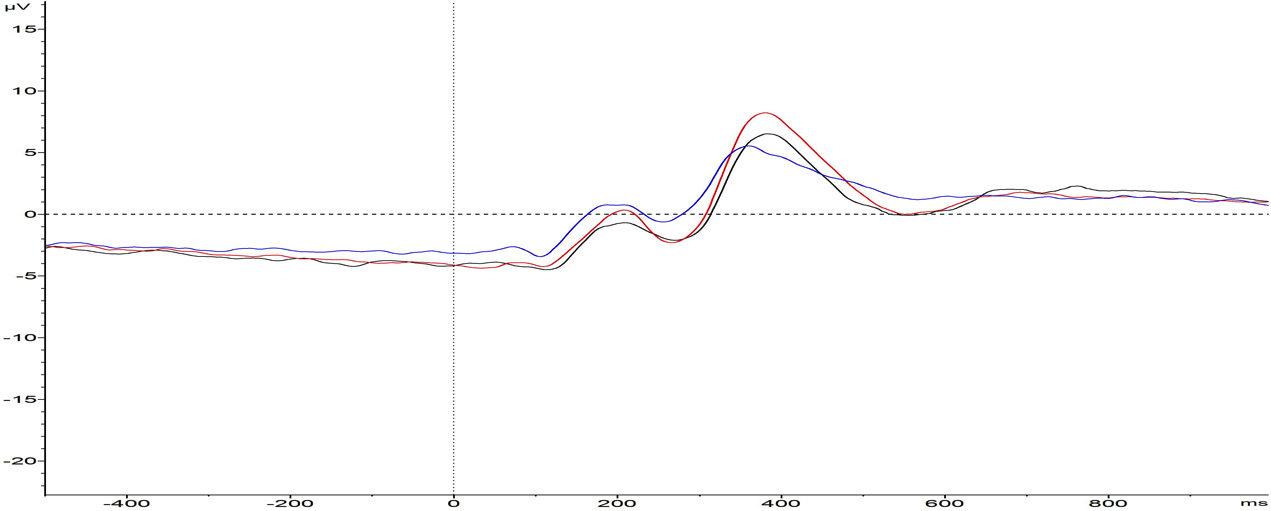


Cz

**Figure S3.** (**A**) Grand average event-related potentials (ERPs) to NoGo stimuli at the FCz (above) and Cz (below) electrodes in the age-matched subsample, showing the NoGo-P3 in the 250-500 ms window (attention-deficit/hyperactivity disorder [ADHD] shown in black; the preterm group shown in blue; and the control group shown in red), and (**B**) topographic maps for each group.
